# Functional neurological disorder treated with psychoeducation: A case report

**DOI:** 10.1097/MD.0000000000041194

**Published:** 2025-01-17

**Authors:** Amal Yousef Alhazmi, Ahmed Ahmed Attar, Suhail Ali Labban, Reema Ghazi Felemban

**Affiliations:** a Psychiatry Section, Department of Medicine, King Abdulaziz Medical City, Ministry of the National Guard Health Affairs, King Abdullah International Medical Research Center, Jeddah, Saudi Arabia; b Department of Neurology, College of Medicine, King Saud Bin Abdulaziz University for Health Sciences, Ministry of the National Guard-Health Affairs, King Abdullah International Medical Research Center, Jeddah, Saudi Arabia; Faculty of Medical Sciences, Department of Medicine, Faculty of Health Sciences, McMaster University, Hamilton, Canada; c College of Medicine, King Saud Bin Abdulaziz University for Health Sciences, King Abdullah International Medical Research Center, Jeddah, Saudi Arabia; d Psychiatry Section, Department of Medicine, King Abdulaziz Medical City, Ministry of the National Guard Health Affairs, King Abdullah International Medical Research Center, College of Medicine, King Saud Bin Abdulaziz University for Health Sciences, Jeddah, Saudi Arabia.

**Keywords:** functional, psychogenic, tremor

## Abstract

**Rationale:**

Psychogenic tremor (PT) is the most common subtype of psychogenic movement disorder, characterized by involuntary movement, and is usually related to occupational injuries or accidents. Psychogenic movement disorder falls under the category of functional neurological disorders, which are diagnosed based on the criteria outlined in the Diagnostic and Statistical Manual of Mental Disorders.

**Patient concerns:**

A 25-year-old Saudi male with a history of recurrent superior ventricular tachycardia presented to the emergency department with tremors affecting all his extremities for 8 days. Tremors were nonrhythmic, continuous, and worsened over time and were exacerbated by reaching objects. There was no history of similar presentations or neurological diseases.

**Diagnoses:**

Examination revealed high-frequency, high-amplitude tremors and rigidity in all extremities, and hyperreflexia in the lower limbs. Laboratory findings were unremarkable; thus, the psychiatric team was consulted for possible PTs.

**Interventions:**

Psychiatric assessments showed no evidence of psychiatric disorders. The patient only received psychoeducation about his diagnosis.

**Outcomes:**

The tremor was completely resolved by the time of discharge.

**Lessons:**

In our case, the patient’s PT resolved entirely with education alone, differing from previous cases that included psychotherapy and medication, emphasizing the importance of the doctor–patient relationship and the need for future research on effective approaches to delivering diagnosis to patients.

## 1. Introduction

Psychogenic movement disorders (PMDs) are characterized by unwanted movements such as tremors, spasms, shaking, or jerks involving any part of the face, neck, trunk, or limbs. PMDs are part of the spectrum of functional neurologic disorders.^[1]^ According to the Diagnostic and Statistical Manual of Mental Disorders (DSM-V) functional neurological disorders are characterized by neurological symptoms, such as motor or sensory dysfunction, that are not better explained by recognized neurological or medical diseases.^[2]^

PMDs are more common in females (72%), and the mean age at first onset was 43 years. The prevalence of psychogenic tremor (PT) is estimated to be 55%, making it the most common form of psychogenic movement disorder, followed by dystonia (39%), myoclonus (13%), tics (6%), gait disorder (3%), and parkinsonism (2%). The onset of PT is usually abrupt. Work-related injuries and motor vehicle accidents are the common precipitating factors. Comorbidities associated with psychiatric disorders, such as depression, and anxiety are also common.^[3]^

The diagnosis of psychogenic tremor is difficult to establish owing to the heterogeneity of presentations in movement disorders and the lack of objective guidelines and parameters for diagnosis, and the separation between organic and psychogenic causes is an ongoing clinical challenge. However, studies have identified some diagnostic features, such as variability in the frequency and amplitude of mass-loading tests in patients with PT. Nevertheless, this sign is not specific and may also be seen in essential tremors and Parkinsonian tremors.^[4,5]^ Another feature is the positive tremor entrainment test, in which a tremor changes when the individual is distracted by imitating the examiner’s rhythmic movements using the opposite hand or foot.^[2,5]^ Another study used functional brain imaging to differentiate between essential tremor and psychogenic tremor based on the distribution of cerebral blood flow. Additionally, differences in the regional metabolic activity of the brain could be an option for differentiating between organic and psychogenic causes.^[6]^ Despite the possibility of using all these methods to diagnose PT, a systematic review by Bartl et al^[5]^ reported that most imaging studies are underpowered and thus may have many false positives, making them unreliable for definitive diagnosis. There was an absence of objective measures and the reliance on clinical findings, which can differ widely among patients highlighted in various case reports in the literature. In this report, we present a case of psychogenic tremor in a 25-year-old male.

## 2. Case presentation

A 25-year-old Saudi male patient had a 6-year history of recurrent supraventricular tachycardia. The patient underwent multiple ablations. The last one was 5 months ago, and he was on bisoprolol 2.5 mg once daily and flecainide 50 mg twice daily. He presented to the emergency department with a history of tremors for 8 days, affecting all his extremities. The tremors were at rest, nonrhythmic, continuous, involuntary, and resolving during sleep but worsened over time. This was the first time the patient experienced such symptoms. There was no prior history of seizures, alteration in the level of consciousness, weakness, head trauma, road traffic accidents, changes in vision, changes in hearing, or substance use. Furthermore, during the past 3 months, he complained of episodes of occipital headache associated with sweating, palpitations, and a systolic blood pressure reading of 220 mm Hg. There was no history of symptoms suggestive of hyperthyroidism, changes in bowel motion, respiratory symptoms, or a family history of endocrine diseases.

Upon examination in emergency department, he was alert and oriented, and the Glasgow Coma Scale score was 15/15. Tremors were characterized by high frequency and high amplitude in all extremities and were exacerbated when reaching objects. The cranial nerves were normal, with symmetrical reactive pupils and normal ocular movements and visual fields. There were no signs of nystagmus, pain, diplopia. The facial muscle strength and sensation remained intact. The upper limb assessment was negative for flapping tremors and pronator drift. The tremor entrainment test was negative. It was challenging to examine the tone in the upper limbs because of tremors and possible rigidity. However, the power remained intact bilaterally. Lower limb examination revealed a rigid tone, with good power observed in hip flexion and extension, bilateral knee flexion at 3/5, and extension at 4/5, with good power in plantar flexion and dorsiflexion. Knee hyperreflexia was noted, with a negative Babinski sign and normal vibration sensation. Coordination tests showed no dysmetria in the finger-to-nose tests but revealed kinetic tremors. The heel-to-shin coordination was equivocal. Imbalance ataxia while standing and toe walking was observed. The results of cardiovascular, respiratory, and abdominal examinations were unremarkable. Upon presentation, his vital signs showed a high blood pressure of 145/104 mm Hg.

After that, the patient was admitted for further investigation of the cause of the tremor. Brain computed tomography and magnetic resonance imaging of the brain, thoracic spine, and spine were unremarkable. Several investigations have been performed including anti-CCP, DNA antibodies, anti-cardiolipin antibodies, antinuclear antibodies, and lupus anticoagulant, to exclude systemic lupus erythematosus and vasculitis. Additionally, thyroid antibodies and thyroid profiles were assessed for thyroiditis and hyperthyroidism. Other tests included ceruloplasmin for Wilson’s disease, hepatitis antibody testing for hepatitis, Syphilis antibody tests, mutation screening for factor V Leiden and prothrombin gene mutations related to thrombophilia, as well as PCR and CSF analysis and cultures for meningitis and encephalitis. An human immunodeficiency virus PCR test was performed to check for the human immunodeficiency virus, and Rheumatoid Factor testing was conducted for rheumatoid arthritis. Tests for anti-GAD, anti-amphiphysin, anti-CASPR/LG1, anti-NMDA receptor antibodies, anti-GABA antibodies, anti-AMPA, Purkinje cells-anti-YO, anti-Hu, B2 glycoprotein I were also done to rule out paraneoplastic diseases. Vitamin levels (B12, E, A, B1, B6, and folate) were evaluated for deficiencies, toxicities, and toxicology screening for substances. All the investigations showed negative results in Tables 1, 2, and 3. Therefore, a psychiatric consultation was sought to explore the possibility of psychogenic tremors.

**Table 1:** Lab investigation of the patient.

Variables		Result	Reference range
Thyroid profile	TSH	0.62	0.6–4.5 mIU/L
	Free T4	13.58	9–19 pmol/L
Complete blood count	Hgb	15.9	13–18 g/dL
	WBC	5.8	4-11 × 10^9^/L
	Basophil	0.06	0-0.01 × 10^9^/L
	Esonphil	0.09	1–0.7 × 10^9^/L
	RBC	5.1	4.5–6.5 × 10^12^/L
	Hematocrit	49.2	40–54%
	MCV	95.6	76–96 fL
	Monocyte	0.4	0.2–0.8 × 10^9^/L
	Lymphcyte	2.02	1.5–4 × 10^9^/L
	Neutrophil	3.14	2–7.5 × 10^9^/L
	MCH	31.0	27–32 pg
	MCHC	32.4	032–36g/dL
	RDW	13.6	11.5–14.5%
	Platelet	370	150–450 × 10^9^/L
Coagulation profile	INR	1.2	0.8–1.2
	PT	14	11–14
	PTT	34	26–41 s
	eGFR	127	60~
Renal profile	CRP	0.3	0–5 mg/L
	Creatinine	68	65–112 µmol/l
	BUN	5.8	2.8–7.4 mmol/L
	BUN	5.8	2.8–7.4 mmol/L
	Potassium	3.8	3.5–4.9 mmol/L
Liver profile	Albumin	48	39–50 g/L
	Total bilirubin	23.2	3.4–22.1 µm mol/L
	Alkaline phosphate	49	39–114 IU/L
	GGT	21	11–68 IU/L
	ALT	15	7–44 IU/L
	AST	16	5–34 IU/L
	Magnesium	0.91	0.72–0.96 mmol/L
Chemistry	Sodium	137	135–144 mmol/L
	Chloride	107	101–111mmol/L
	Phosphorus	1.00	0.8–1.44 mmol/L
	Calcium	2.36	2.11–2.56 mmol/L
	Cannabinoids	Negative	
Toxicology screen panel	Barbiturate	Negative	
	Benzodiazepine	Negative	
	Cocaine	Negative	
	Opiate	Negative	

ALT = alanine transaminase, AST = aspartate transaminase, BUN = blood urea nitrogen, CRP = C-reactive protein, eGFR = estimated glomerular filtration rate, GGT = gamma-glutamyl transferase, Hgb = hemoglobin, INR = international normalized ratio, MCV = mean corpuscular volume, PT = protheombin time, PTT = partial thromboplastin time, RBC = red blood cells, TSH = thyroid stimulating hormone, WBC = white blood cells.

**Table 2:** Autoimmune antibody investigation of the patient.

Variable		Result	Reference range
Anti-CCP IgG antibody		0.93	<20 units is negative
DNA antibody, double-stranded		14.97	<200 IU/mL is negative
Anti-SS-A		1.88	<20 units is negative
Anti-SS-B		2.78	<20 units is negative
Anti-cardiolipin antibody (ACA)	ACA IgM	7.40	<12 MPL units is negative
	ACA IgA	3.37	<12 APL units is negative
	ACA IgG	0.74	<12 GPL units is negative
Antinuclear antibody (ANA) immunofluorescence assay		Negative	
Rheumatoid factor		<10.90	<15.9 IU/mL is negative
Lupus anticoagulant	Lupus anticoagulant 1	39	26–44 s
	Lupus result	Absent	
Thyroid antibody	C4 complement	0.21	0.1–0.4 g/L
	C3 complement	1.14	0.9–1.8 g/L
	TPO antibody	2.72	1–16 IU/mL
	Thyroglobulin antibody	<5.00	5–100 IU/mL
	Thyroglobulin level	8.9	0.2–70 ng/mL
	P-ANCA (MPO)	5.77	<20 units is negative
	C-ANCA (PR3)	2.60	<20 units is negative
	TSH receptors binding antibody	<1	<OR = 2 IU/L
Syphilis total antibody		Negative	
FII & FVL mutation	FII mutation	Normal	
	FII mutation interpretation	Not detected	
	FVL mutation	Normal	
	FVL mutation interpretation	Not detected	
Meningitis/encephalitis panel multiplex PCR	Human parechovirus	Not detected	
	Streptococcus pneumoniae	Not detected	
	Herpes simplex virus-1	Not detected	
	Human herpes virus-6	Not detected	
	Listeria monocytogenes	Not detected	
	Herpes simplex virus-2	Not detected	
	Varicella zoster virus	Not detected	
	Cytomegalovirus	Not detected	
	Escherichia coli K1	Not detected	
	Haemophiles influenzae	Not detected	
	Neisseria meningitidis (encapsulated)	Not detected	
	Streptococcus agalactiae	Not detected	
	Enterovirus	Not detected	
	Meningitis/encephilitis Multiplex PCR	Not detected	
	Cryptococcus neoformans/gattii	Not detected	
CSF analysis and culture	CSF TP	0.26	0.15–0.45 g/L
	CSF glucose	3.2	2.2–3.9 mmol/L
	Cerebrospinal fluid culture	Negative	
Hepatitis antibody/antigen	Hepatitis C antibody	Negative	
	HBsAg	Negative	
	AntiHbc	Negative	
	AntiHbs	0.00	< 10 mIU/L not detected
Anti-GAD		<10.00	< 10 IU/mL is negative
Anti-amphiphysin		Negative	
Anti-CASPR/LG1		Negative	
Anti-NMDA receptor antibodies		Negative	
Anti-GABA antibodies		Negative	
Anti-AMPA		Negative	
Purkinje cells-anti YO		Negative	
Anti-Hu		Negative	
Anti-GABA antibodies		Negative	
B2 glycoprotein I	B2 glycoprotein I IgA	2.41	<20 SAU is negative
	B2 glycoprotein I IgG	0.82	<20 SGU is negative
	B2 glycoprotein I IgM	1.65	<20 SMU is negative

ACA = anti-cardiolipin antibody, ANA = antinuclear antibody, ANCA = perinuclear anti-neutrophil cytoplasmic antibodies, anti-AMPA = α-amino-3-hydroxy-5-methyl-4-isoxazolepropionic acid, anti-CASPR2 = contactin-associated protein-like 2, anti-CCP = anti-cyclic citrullinated peptides, anti-GABA = antibodies against gamma-aminobutyric acid, anti-GAD = antibodies to glutamic acid decarboxylase, anti-HBc = antibody to hepatitis B core, antiHbs = antibody to hepatitis B surface antigen, anti-LGI1 = leucine-rich glioma inactivated 1, anti-NMDA = antibodies against the N-methyl-D-aspartate, anti-SSA = anti–Sjögren’s-syndrome-related antigen, APL = IgA phospholipid units, CSF = cerebrospinal fluid, DNA = deoxyribonucleic acid, FII & FVL = factor V Leiden, GPL = IgG phospholipid units, HBsAg = hepatitis B surface antigen, HBsAg = hepatitis B surface antigen, IgA = immunoglobulin A, IgG = immunoglobulin, IgM = immunoglobulin M, MPL = IgM phospholipid units, PCR = polymerase chain reaction, SAU = standard IgA anti-beta 2 glycoprotein 1 units, SGU = standard IgG anti-beta 2 glycoprotein 1 units, SMU = standard IgM anti-beta 2 glycoprotein 1 units, TPO = thyroid peroxidase.

**Table 3:** Vitamins investigation of the patient.

Lab	Result	Reference range
Vitamin B6	33	5–50 µg/L
Vitamin K1	<200	320–1000 ng/L
Vitamin B2	279	180–295 µg/L
Vitamin E	11.5	5–200 mg/L
Vitamin A	696	300–700 µg/L
Vitamin B1	60	25–85 µg/L
Vitamin B12	285.0	135–455 pmol/L
Ceruloplasmin	0.20	0.22–0.58 g/L
Folate	15.50	6.4 nmol/L

Upon psychiatric evaluation, the patient denied any history suggestive of major depressive disorder, mania, hallucinations, delusions, generalized anxiety, social phobia, panic attacks, obsessive-compulsive disorder, illness anxiety disorder, smoking, or substance use disorder. Moreover, there was no history of suicidal or homicidal thoughts or physical, sexual, or verbal abuse. He also denied a history of recent stressors. The patient had never been admitted to a mental health hospital and had not taken any psychotropic medication. The patient had no family history of psychiatric disorders or similar presentations. His relationship with his family and self-hygiene were good.

Mental status examination revealed a young male who looked at his stated age. He had an average body build with good grooming and hygiene. He was cooperative and had good eye contact. The speech assessment was coherent, relevant, good amount, normal rate, and normal tone. His mood was euthymic, and his effect was reactive and consistent with his mood. Regarding thought process and content, the patient did not have formal thought disorder, suicidal thoughts, homicidal ideation, delusions, obsessions, or hallucinatory attitudes during the interviews. The patient was alert and oriented to the time, place, and person. He had good judgment; however, he did not believe that this symptom was due to psychiatric illness.

The patient was admitted for 6 days, he received only his home cardiac medications which are bisoprolol and flecainide. The psychiatric team provided psychoeducation about the diagnosis and the prognosis of functional neurological disorder to the patient and his family. No psychotherapy sessions were held, and no psychotropic medications were administered. The tremor subsided during admission, and he was discharged on the same cardiac medications.

## 3. Discussion

Distinguishing between functional and organic movement disorders is challenging. Psychogenic tremors are characterized by changes in frequency and amplitude during distractibility maneuvers.^[1]^ In 1 study, 4 cases of Tourette syndrome were misdiagnosed as psychogenic tremors. The reason for these misdiagnoses was attributed to the similarities between both conditions, such as the bizarre movements that increase with stress and improve with distractions, the presence of associated psychopathological disorders, and periods of spontaneous remission.^[7]^ In our case, the tremor did not change in frequency and amplitude during the distractibility maneuver. This highlights the need for objective measurements rather than subjective measurements.

The underlying mechanisms of psychogenic tremors are poorly understood. Psychological factors may play a role in the development of psychogenic tremors.^[3]^ Patients with functional neurological disorders experience higher levels of disability and distress than those with organic neurological diseases in an outpatient setting.^[8]^ In our case, the patient had no recent life stressors, trauma, or other psychiatric comorbidities. This emphasizes that functional neurological disorders can arise independently of life stressors and are multifactorial.

Given the complex nature of psychogenic tremors, there is heterogeneity in their treatment. In 1 case report, severe psychogenic tremor of both wrists in a 13-year-old girl was successfully treated with a customized wrist brace, cognitive behavior therapy, and positive feedback through the use of video recordings of improvement, which increased the patient’s motivation. The severity of the tremors was reduced by 80% upon discharge. Two weeks after discharge, the tremor completely resolved.^[9]^ Additionally, a study conducted among patients with functional neurological tremors revealed an increased activation of the anterior cingulate/paracingulate regions during emotional task processing. This activation decreased after a 12-week course of cognitive behavioral therapy.^[10]^ In addition to cognitive behavioral therapy, psychodynamic therapy has been studied in PMD. In a study conducted among patients with PMD, most participants were middle-aged females who underwent treatment with psychodynamic psychotherapy, revealing a 60% improvement in symptoms. The mean number of therapy sessions was 4.9.^[11]^

The evidence of pharmacological treatment for PMD is still limited due to the lack of randomized controlled trials. In a case report, a 38-year-old male patient was diagnosed with psychogenic tremor as a conversion reaction after he had lost his job. He was referred for psychological counseling and was started on escitalopram 20 mg once a day. Four months later, he showed significant improvement in his tremor. After another 4 months, he had been rehired at his previous job, and his tremor was completely resolved.^[3]^ In our case, neither pharmacological treatment nor psychotherapy sessions were provided. The patient and his family were educated about the diagnosis of PT and the mind-body connection. The tremor completely resolved after 6 days of hospitalization.

Sharing the diagnosis with the patient is a crucial part of the management. Clinicians may feel frustrated by the lack of definitive investigations, which may lead to avoidance of discussing the psychogenic tremor diagnosis with patients. Additionally, patients may be reluctant to accept the diagnosis and negative test results. These factors can delay a clear diagnosis and worsen the prognosis. A retrospective study assessed the 3-year long-term outcomes of 127 patients diagnosed with PT, and it showed that 56.6% of patients reported improvement in their tremors. The factors that predicted a favorable outcome were the elimination of stressors and patients’ perception of effective treatment by their physician.^[12]^ Thus, the clinician should establish a strong rapport and help the patients to accept the diagnosis.

## 4. Conclusion

In our case, the patient received only education regarding his diagnosis of psychogenic tremor, and the tremor resolved completely. This contrasts with previous case reports that involved psychotherapy sessions and pharmacological treatment. The doctor-patient relationship plays a key role in the management of psychogenic tremors, and this emphasizes the need for future research on effective strategies for delivering the diagnosis to patients.

## Author contributions

**Investigation:** Amal Yousef Alhazmi, Reema Ghazi Felemban.

**Methodology:** Amal Yousef Alhazmi, Suhail Ali Labban.

**Supervision:** Ahmed Ahmed Attar, Reema Ghazi Felemban.

**Writing – original draft:** Amal Yousef Alhazmi, Ahmed Ahmed Attar, Suhail Ali Labban, Reema Ghazi Felemban.

**Writing – review & editing:** Amal Yousef Alhazmi, Ahmed Ahmed Attar, Suhail Ali Labban, Reema Ghazi Felemban.
